# Versatile graceful degradation framework for bio-inspired proprioception with redundant soft sensors

**DOI:** 10.3389/frobt.2024.1504651

**Published:** 2025-01-06

**Authors:** Taku Sugiyama, Kyo Kutsuzawa, Dai Owaki, Elijah Almanzor, Fumiya Iida, Mitsuhiro Hayashibe

**Affiliations:** ^1^ Neuro-robotics Laboratory, Department of Robotics, Graduate School of Engineering, Tohoku University, Sendai, Japan; ^2^ Bio-Inspired Robotics Laboratory, Department of Engineering, University of Cambridge, Cambridge, United Kingdom

**Keywords:** soft sensors and actuators, redundant sensors, neural network, self-adaptation, proprioception, graceful degradation

## Abstract

Reliable proprioception and feedback from soft sensors are crucial for enabling soft robots to function intelligently in real-world environments. Nevertheless, soft sensors are fragile and are susceptible to various damage sources in such environments. Some researchers have utilized redundant configuration, where healthy sensors compensate instantaneously for lost ones to maintain proprioception accuracy. However, achieving consistently reliable proprioception under diverse sensor degradation remains a challenge. This paper proposes a novel framework for graceful degradation in redundant soft sensor systems, incorporating a stochastic Long Short-Term Memory (LSTM) and a Time-Delay Feedforward Neural Network (TDFNN). The LSTM estimates readings from healthy sensors to compare them with actual data. Then, statistically abnormal readings are zeroed out. The TDFNN receives the processed sensor readings to perform proprioception. Simulation experiments with a musculoskeletal leg that contains 40 nonlinear soft sensors demonstrate the effectiveness of the proposed framework. Results show that the knee angle proprioception accuracy is retained across four distinct degradation scenarios. Notably, the mean proprioception error increases by less than 1.91°(1.36%) when 30% of the sensors are degraded. These results suggest that the proposed framework enhances the reliability of soft sensor proprioception, thereby improving the robustness of soft robots in real-world applications.

## 1 Introduction

Soft sensors are crucial technologies in soft robotics. They enable soft robots’ intelligent autonomy by providing sensory feedback (Hegde et al., 2023). Soft sensors offer various sensing modalities, such as strain, tactile sensation, and temperature. Among these sensing modalities, the awareness of a robot’s own shape, which is called proprioception in neuroscience, is particularly important (Wang et al., 2018; Yang et al., 2024). Reliable proprioception is vital for the functionality of soft robots in an unstructured environment, as the failure in proprioception impairs their autonomous capabilities (Yang et al., 2024). Due to their softness, soft robots can experience significant and nonlinear deformations in response to a control input (Polygerinos et al., 2017; Yasa et al., 2023). Hence, soft robot modeling is generally challenging, and minor discrepancies between the model and the actual robot can deteriorate open-loop control accuracy (Wang et al., 2018). Furthermore, soft robots are prone to passive deformation. For these reasons, reliable proprioception feedback is crucial for soft robots to adapt to continuous deformation and maintain autonomy for effective task performance. (Wang et al., 2018; Lin Z. et al., 2023; Chen et al., 2018; Thuruthel et al., 2019; Kawaharazuka et al., 2022). For example, prior work by Alatorre et al. (2022) reported the use of proprioceptive feedback for a soft continuum robot improved the closed-loop position control accuracy by 65%. Indeed, many review papers have highlighted the importance of consistently reliable proprioception for soft robots (Hegde et al., 2023; Terryn et al., 2021; Yang et al., 2024; Lin Z. et al., 2023).

However, soft sensors are susceptible to various damages (Terryn et al., 2021), interfacial debonding (i.e., wiring failure) (Lo Preti et al., 2022), and fatigue (Roels et al., 2022). Nevertheless, in real-world applications like fruit harvesting and rescue (Wang et al., 2018), soft robots are exposed to multiple sources of damage. Also, soft robots generally undergo repeated large deformation. As a result, soft sensors experience degradations (i.e., failures) that distort sensor signals (Yang et al., 2024). In reality, some studies have reported sensor failures during soft robot applications (Lin Y.-H. et al., 2023; Roels et al., 2022).

To overcome the fragility of soft sensors and enhance the reliability of proprioception, researchers have utilized self-healing materials (Khatib et al., 2021; Terryn et al., 2021; Mazzolai et al., 2022) and redundant soft sensor configurations (Thuruthel et al., 2019; Kawaharazuka et al., 2022; Wang et al., 2023). Then, by leveraging learning-based approaches, researchers have recovered or retained proprioception accuracy despite sensor failure. Note that a learning-based approach is popular to model soft sensors (Kim et al., 2021), as they present numerous modeling challenges (Polygerinos et al., 2017).

As the review paper describes (Khatib et al., 2021), researchers have utilized self-healing materials to fabricate soft sensors (the Supplementary Material provides an example). Yet, most self-healing materials require minutes or even hours to complete the healing process, resulting in downtime and low sensing frequency (Yang et al., 2024). Additionally, a healing process will change sensor properties. Thus, recalibration is required to recover proprioception accuracy after sensor degradations.

On the other hand, redundant soft sensors realize instant adaptation to degradations and failures without downtime or intervention [e.g., recalibration (Roels et al., 2022), reconfiguration of sensor position (Nguyen and Ho, 2022)]. Due to redundancy, healthy sensors can compensate for the other ones and retain proprioception accuracy. This specific property is called Graceful Degradation (Thuruthel et al., 2019), which is essential for achieving consistently reliable proprioception without downtime. Here, redundancy refers to having multiple sensors that provide overlapping or similar information, akin to biological sensory systems (Proske and Gandevia, 2012). For example, animals possess redundant muscle spindles within muscle groups to perceive their joint angles (Erin et al., 2016), enabling adaptation to changes in musculoskeletal configuration (Philipp et al., 2023). By leveraging such a biological sensory system, soft robot sensing can be more robust against damage (Thuruthel et al., 2019) and provide feedback of multiple sensory modalities over a large sensing area (Hardman et al., 2023).

Graceful degradation in soft sensors has been typically realized using neural networks (Thuruthel et al., 2019; Wang et al., 2023). For example, Thuruthel et al. (2019) realized accurate multimodal sensing of a soft continuum actuator by combining a redundant sensor configuration (three embedded soft sensors and one pressure sensor) with an LSTM network. In addition to their main contribution, the researchers demonstrated that the LSTM network adapted to the virtual loss of one or two soft sensors and retained proprioception accuracy in the simulation experiment. Some researchers have implemented graceful degradation for soft sensor exteroception and multimodal sensing. Supplementary Material provides other examples of graceful degradation. Yet, the proposed methods did not incorporate the detection mechanism for sensor degradation, and the evaluation scenarios were limited to the complete sensor loss (i.e., zeroing sensor readings). In contrast, soft sensors can experience diverse degradations during operation due to their softness and nonlinearity: cut, partial breakage, plastic deformation, length deviation due to interferences, and even temperature and humidity affect sensor readings (Terryn et al., 2021; Khatib et al., 2021; Porte et al., 2024; Terryn et al., 2022; Shen et al., 2016). Without detecting sensor degradation, a non-zero but distorted sensor reading will significantly affect the proprioception process and decline the accuracy.

**FIGURE 1:**
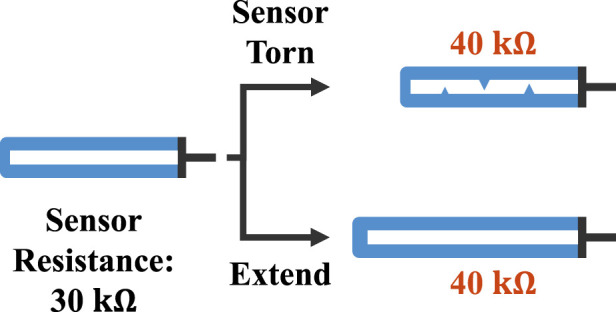
A simple example of non-unique mapping. One resistive soft sensor (white) is embedded in a pressure-driven soft extending actuator (blue) with tubings (black). When the sensor is partially torn, its resistance typically increases (Hegde et al., 2023). Consequently, the distorted sensor readings (upside) become identical to those of a healthy sensor with a longer actuator length (bottom), leading to non-unique mapping.

Therefore, detecting and localizing various types of soft sensor degradation is essential for achieving graceful degradation. To our best knowledge, no study has addressed such graceful degradation for soft sensor proprioception. A few researchers implemented it for soft tactile sensing (Lo Preti et al., 2022) and multimodal sensor data fusion (Lee et al., 2021). Supplementary Material provides the details of these studies. However, applying the fault detection method utilized in these studies to soft sensor proprioception is difficult due to a non-unique mapping of a soft sensor (Thuruthel et al., 2019; Hegde et al., 2023) and the difficulty in training data collection. *Non-unique mapping* in this paper indicates that sensor degradation results in similar or identical sensor readings for different system states (Figure 1). This non-unique mapping makes signal-based fault detection (Lo Preti et al., 2022) inapplicable. On soft sensor proprioception, unlike tactile sensing, it is impracticable to distinguish whether the signal variation is due to a deformation of a proprioception target or due to sensor degradation. Regarding the fault detection through reconstruction (Lee et al., 2021), extensive pre-training of degradation pattern is required for precise fault detection; however, the infinite number of soft sensor failure modes makes the training data acquisition infeasible. Additionally, the non-unique mapping would affect fault detection accuracy. Sensor degradation will be overlooked if the distorted sensor readings are similar to healthy sensor readings in different states of a target, leading to reduced proprioception accuracy.

This paper proposes a novel learning-based framework for graceful degradation, enabling redundant soft sensors to maintain reliable proprioception under various sensor degradation. To the authors’ best knowledge, this is the first framework to achieve comprehensive graceful degradation for redundant soft sensors by implementing fault detection that avoids non-unique mapping and impracticable dataset preparation. The proposed framework consists of a stochastic LSTM for sensor fault detection and a Time-delay Feedforward Neural Network (TDFNN) for proprioception. The LSTM receives control inputs to a proprioception target and outputs the corresponding values of healthy sensors. The actual sensor readings are then compared with the estimates. Next, statistically unreliable values are identified as degraded and zeroed. This fault detection procedure prevents non-unique mapping from occurring. This procedure also realizes dataset preparation without seminal characterization and inclusion of all possible sensor failure modes. Subsequently, the TDFNN receives the processed sensor data and performs proprioception. As a result, the proposed framework realizes the comprehensive graceful degradation to diverse degradation of redundant soft sensors. Our framework was evaluated by simulation experiments with a nonlinear musculoskeletal leg model that contains 40 nonlinear soft sensors. We utilized a musculoskeletal system because a realistic and reliable simulation model is available, while it shares similar characteristics with soft robots (Polygerinos et al., 2017; Driess et al., 2018; Masuda et al., 2019; Carpenter, 1968; Hirashima and Oya, 2016; Almanzor et al., 2023). We demonstrate that the proposed framework retains proprioception accuracy against four different degradation scenarios. Then, we show that the framework can tolerate degradation in more than half of all sensors. Finally, we present the framework’s scalability with two additional musculoskeletal leg models featuring different numbers of sensors or muscle-joint configurations.

## 2 Methods

### 2.1 Architecture of the proposed framework

#### 2.1.1 Architecture overview

**FIGURE 2:**
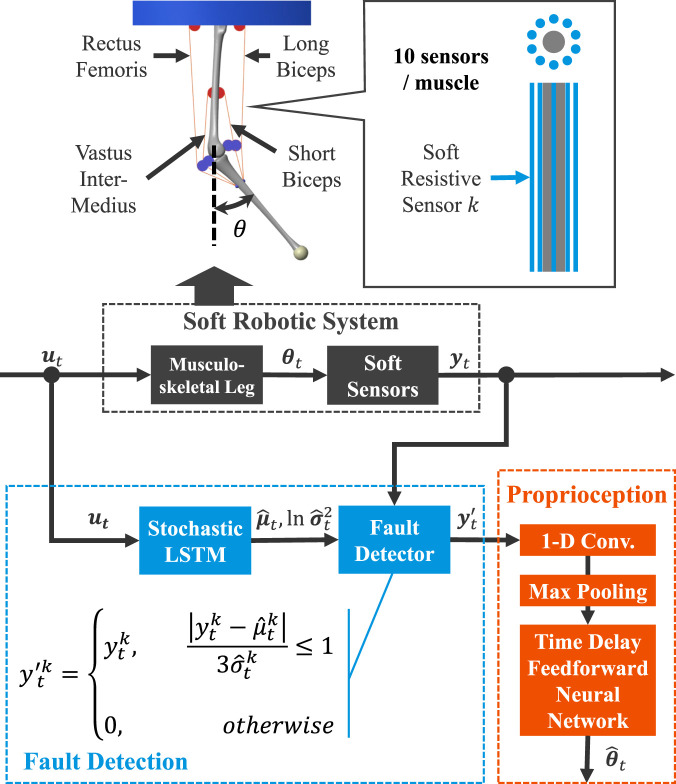
The simulation testbed and architecture of the proposed framework. Supplementary Figure S1 in the supplemental material provides the detailed process flow. The $u_{t}$ , $\theta_{t}$ , and $y_{t}$ represent the control input, the target state for proprioception, and the sensor readings at time step t . The LSTM is responsible for fault detection, while the TDFNN performs proprioception. First, the LSTM zeroes out statistically abnormal sensor readings. These processed readings are then input to the TDFNN, which outputs the estimated states. As a result, the framework achieves graceful degradation and realizes reliable proprioception despite diverse degradation in the constituent soft sensors.

Figure 2 describes the process flow of the proposed framework at the time step t . The LSTM continuously receives control input $u_{t}$ . Then, the LSTM estimates the current values of healthy sensors ${\hat{y}}_{t}$ . The outputs of the LSTM are the estimated mean ${\hat{\mu }}_{t}$ and variance ${\hat{\sigma }}_{t}^{2}$ of ${\hat{y}}_{t}$ which are assumed to follow the normal distribution. Next, the anomaly coefficient $A_{t}$ of sensor k is calculated with actual sensor readings $y_{t}$ :

$$A_{t}^{k}=\frac{\left|y_{t}^{k}-{\hat{\mu }}_{t}^{k}\right|}{3{\hat{\sigma }}_{t}^{k}}$$ (1)

If $A_{t}^{k}>1$ , the sensor k is identified as degraded, and $y_{t}^{k}$ is zeroed since it is statistically 99.7% abnormal. Finally, the TDFNN receives 20 steps of processed sensor readings $y_{t-20:t}^{\prime }$ and outputs the estimated states ${\hat{\theta }}_{t}$ .

In this study, the values from abnormal sensors are processed to zero. Unifying abnormal values obviates the need to prepare datasets for every possible sensor degradation scenario and prevents non-unique mappings. Moreover, using zero facilitates the generalization of the TDFNN with the max pooling layer. This processing is further justified by the baseline resistance of soft sensors, which is always greater than zero unless a sensor rupture occurs.

#### 2.1.2 Stochastic LSTM for fault detection

The stochastic LSTM is responsible for real-time fault detection and acts as a healthy forward model. The LSTM accurately estimates healthy sensor values by accounting for the dynamic nonlinearities of the proprioceptive target. The LSTM is implemented as a stochastic model which outputs ${\hat{\mu }}_{t}$ and ${\hat{\sigma }}_{t}^{2}$ instead of ${\hat{y}}_{t}$ (Murata et al., 2013). The threshold for the direct comparison between $y_{t}$ and ${\hat{y}}_{t}$ is affected by the actuation speed of a proprioception target. If the target is not actuating, the variation of u is close to zero, which leads to the smaller variation of y . In such situations, even the slight differences between y and $\hat{y}$ are more likely to be due to sensor degradation. Thus, the threshold has to be small if the target actuates slowly and large otherwise to maintain fault detection accuracy. The anomaly coefficient A , derived using the variance predicted by the stochastic LSTM, removes the need for dynamic threshold adjustments. Consequently, accurate and consistent quantitative fault detection is achieved, regardless of the actuation state of a proprioception target or parameter tuning.

The stochastic LSTM is trained using a loss function [Equation 3, (Murata et al., 2013)] that is derived as follows. First, the current reading from sensor k , $y_{t}^{k}$ are assumed to follow the normal distribution. The probability density function of the normal distribution $N^{k}$ is written as:

$$N\left(y_{t}^{k};\mu_{t}^{k},\sigma_{t}^{k}\right)=\frac{1}{\sqrt{2\pi {\left(\sigma_{t}^{k}\right)}^{2}}}\exp\left(-\frac{{\left(y_{t}^{k}-\mu_{t}^{k}\right)}^{2}}{2{\left(\sigma_{t}^{k}\right)}^{2}}\right)$$ (2)

where $\mu_{t}^{k}$ and ${\left(\sigma_{t}^{k}\right)}^{2}$ are the mean and variance of the distribution. The maximum likelihood estimation of $N^{k}$ yields optimized ${\hat{\mu }}_{t}^{k}$ and ${\left({\hat{\sigma }}_{t}^{k}\right)}^{2}$ . A function L is obtained by converting the Equation 2 into a negative log-likelihood and removing constant:

$$L\left({\hat{\mu }}_{t},{\hat{\gamma }}_{t}\right)=\frac{1}{n}\overset{n}{\underset{k=1}{\sum }}\left({\hat{\gamma }}_{t}+\frac{{\left(y_{t}-{\hat{\mu }}_{t}\right)}^{2}}{\exp\left({\hat{\gamma }}_{t}\right)}\right)$$ (3)

where n is the total number of sensors and ${\hat{\gamma }}_{t}=\ln\left({\hat{\sigma }}_{t}^{2}\right)$ . The estimated variance ${\hat{\sigma }}_{t}^{2}$ is output in the form of ${\hat{\gamma }}_{t}$ to avoid gradient explosion. The training of the LSTM minimizes L , which is equivalent to the maximum likelihood estimation of N . Consequently, the LSTM outputs maximum likelihood ${\hat{\mu }}_{t}$ and ${\hat{\sigma }}_{t}^{2}$ after the training.

#### 2.1.3 FNN for proprioception

The TDFNN is responsible for proprioception. The TDFNN outputs the estimated states ${\hat{\theta }}_{t}$ , receiving processed time-series sensor values. We incorporated a time-delay architecture to address the delays typical of soft sensors. For graceful degradation, a proprioception network needs to learn and adapt to the temporal variations of the healthy/zeroed sensor combination. Using a static network enables a more efficient training process and reduces training time. Additionally, in terms of real-time proprioception, this approach avoids the time-consuming sequential estimation with LSTMs.

The combination of healthy and zeroed sensor data increases quadratically. Thus, as the number of sensors in a single soft actuator increases, it becomes infeasible for a simple TDFNN to learn all possible combinations. We addressed this issue using a 1-D convolution and max pooling layers before fully-connected layers. The convolution across adjacent sensors and max pooling extract features for accurate proprioception, ignoring zeroed sensor readings. This approach enhances translation invariance. Also, augmented training data enables more efficient training (Section 2.3).

### 2.2 Simulation experiment setup

**FIGURE 3:**
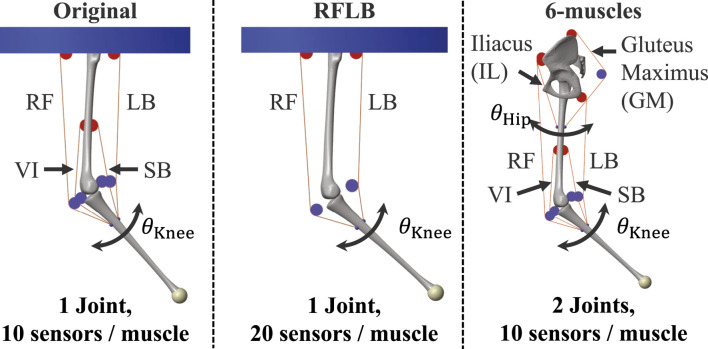
Musculoskeletal leg models used in the experiments.

We utilized a musculoskeletal leg simulation to evaluate the proposed framework because a realistic nonlinear muscle model is available (Hill, 1938). The leg model was constructed on MATLAB/Simscape. The proprioception target state was a joint angle of the leg θ (Figures 2, 3). Soft sensors measured muscle length. Humans perceive joint angles through signals from muscle spindles within their muscles (Erin et al., 2016). Muscle spindles generate sensory signals via primary and secondary afferent fibers. They can be approximated as muscle velocity and length, respectively (Marques et al., 2014). Using the nonlinear musculoskeletal leg and soft sensor model for such proprioception enables simulation based on a reasonable model, and it is similar to soft robot proprioception with embedded soft sensors (Thuruthel et al., 2019). Additionally, the musculoskeletal leg model shares similar characteristics with soft robots, such as static and dynamic nonlinearity (Sugiyama et al., 2024; Polygerinos et al., 2017; Driess et al., 2018; Masuda et al., 2019) and the lack of a unique solution to achieve the desired robot state (i.e., motor equivalence problem) (Carpenter, 1968; Hirashima and Oya, 2016; Almanzor et al., 2023). Besides, prior studies on proprioception with a redundant sensor configuration also utilized musculoskeletal systems (Kawaharazuka et al., 2022; Thuruthel et al., 2020).

We modeled the sensors as soft resistive strain sensors (Souri et al., 2020). The model was built based on literature that characterized a soft resistive sensor made from a carbon nanocomposite elastomer (Muth et al., 2014). Note that it is one of the most commonly used materials for such sensors (Yamada et al., 2011; Thuruthel et al., 2019). Soft sensors typically exhibit nonlinear responses due to elastomeric materials, temporal nonlinearity such as response delay, and individual differences (Hegde et al., 2023; Terryn et al., 2022; Thuruthel et al., 2021; Sugiyama et al., 2021). Hence, we applied a second-order delay to the sensor length changes and modeled the strain-resistance characteristics with a quadratic function. Moreover, individual differences were implemented by randomizing the parameters required for these modelings. Finally, we added Gaussian noise for a more realistic simulation (Lin Y.-H. et al., 2023). Through this process, we simulated the typical response and temporal nonlinearities of soft resistive strain sensors made from carbon elastomer. These nonlinearities are also observed for the other sensor materials, such as liquid metal and hydrogel (Park et al., 2012; Shi et al., 2021; Lu et al., 2020; Guan et al., 2023; Cai et al., 2017; Shen et al., 2022; Xu et al., 2019; Wang et al., 2016).

#### 2.2.1 Musculoskeletal leg model

Figure 3 (*Original* model) describes the configuration of the model. The leg consists of the femur and tibia. The angle of the knee joint θ was set as a proprioception target. The joint was simulated as a revolute joint and actuated by two pairs of agonist-antagonist muscles: the Rectus Femoris (RF) and the Long Biceps (LB), the Vastus Intermedius (VI) and the Short Biceps (SB). The range of motion was 0°to 140° . The positioning of the muscles was based on the literature (Marques et al., 2014; Geyer and Herr, 2010). The femur was suspended from the ceiling and simulated as a weld joint. The masses of both femur and tibia were 1 kg. Each muscle approximated a muscle group containing muscle spindles (Erin et al., 2016). The muscles were modeled as Hill-type muscles (Hill, 1938) consisting of an active contractile, a passive spring, and a passive damper element arranged parallelly (Almanzor et al., 2024). Consequently, a muscle j output nonlinear force according to muscle activation signal $a^{j}\left(t\right)$ while possessing nonlinear passive dynamics (Equation 4):

$${F\;}_{\mathrm{MTU}}^{j}\left({\hat{l\;}}^{j}\left(t\right),{\hat{v\;}}^{j}\left(t\right),{a\;}^{j}\left(t\right)\right)={F\;}_{CE}^{j}\left({\hat{l\;}}^{j}\left(t\right),{\hat{v\;}}^{j}\left(t\right),{a\;}^{j}\left(t\right)\right)+{F\;}_{\mathrm{spring}}^{j}\left({\hat{l\;}}^{j}\left(t\right)\right)+{F\;}_{\mathrm{damper}}^{j}\left({\hat{v\;}}^{j}\left(t\right)\right)$$ (4)

where $\hat{l}\left(t\right)$ is the muscle length normalized to the initial resting length $l_{\mathrm{ref}}$ , and $\hat{v}$ is the muscle contraction velocity that is normalized to the maximum contraction velocity $v_{\mathrm{ref}}$ . The contractile element receives a(t) and generates the active contraction force $F_{CE}$ , which incorporates a normal distribution function and a sigmoid function to approximate the natural activation of muscles (Meyer et al., 2017; Heinen et al., 2016; Haeufle et al., 2014). The spring and damper elements generate the passive forces of the muscle, where the spring force $F_{\mathrm{spring}}$ depends on muscle length, and the damper force $F_{\mathrm{damper}}$ depends on muscle velocity. These elements simulate the natural behavior of real muscles. Muscles tend to contract back to their resting length when stretched by external forces and oppose the speed of extension or contraction (Haeufle et al., 2014). The detailed implementation of the model was described in the authors’ previous work (Almanzor et al., 2024). Table 1 lists the parameters of each muscle required to build the model: the maximum isometric force $F_{\text{MAX}}$ [N] derived from Chou-Hannaford equation (Chou and Hannaford, 1996), the initial resting length $l_{\text{ref}}$ [m], and empirically found maximum contraction velocity $v_{\text{ref}}$ [m/s].

**TABLE 1:** The parameters of the musculoskeletal leg models.

		RF	LB	VI	SB	IL	GM
$F_{\text{MAX}}$	Original	1,335	1,315	302	305	—	—
	6-muscles	2,378	940	384	200	72	661
$l_{\text{ref}}$	Original	0.46	0.45	0.27	0.27	—	—
	6-muscles	0.59	0.39	0.29	0.24	0.21	0.35
$v_{\text{ref}}$	Original	0.23	0.32	0.022	0.029	—	—
	6-muscles	1.31	0.45	0.021	0.028	0.018	0.20

For the scalability evaluation of the proposed framework in Section 3.3, two other musculoskeletal leg models, *RFLB* and *6-muscles*, were built. Figure 3 shows the configuration of each model. The RFLB model was built by removing VI and SB muscles from the original model. The knee joint was actuated only by RF and LB muscles. This model shares the same parameters as the original model. The 6-muscles model was cited from the authors’ previous work (Almanzor et al., 2024) and consisted of the pelvis, femur, and tibia. Six muscles, RF, LB, VI, SB, Iliacus (IL), and Fluteus Maximus (GM), were attached to actuate the hip and knee joint. The pelvis was fixed in the air as a weld joint. For both models, the range of motion for the knee angle was 0° to 140° , and that for the hip angle was −70° to 70° .

#### 2.2.2 Soft resistive sensor model

**FIGURE 4:**
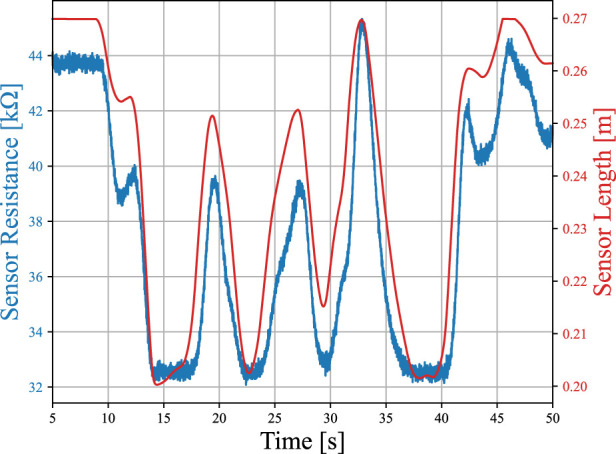
Example of simulated sensor response (blue) and corresponding sensor length (red). This sensor was attached to the Short Biceps (SB).

A soft resistive strain sensor k on a muscle j measures simulated muscle lengths (i.e., sensor length) $l^{j}\left(t\right)$ [m] as resistance $R^{j,k}\left(t\right)$ [kΩ]. We modeled the sensor as follows so that the model approximates the length-resistance characteristics and the response delay experimentally verified by Muth et al. (2014). Note that other studies that characterized the properties of carbon elastomer soft strain sensors also reported similar nonlinearities (Park et al., 2019; Yamada et al., 2011; Li et al., 2015; Mattmann et al., 2008; Shintake et al., 2018). Figure 4 illustrates an example of simulated sensor response and corresponding sensor length.

First, sensor length was converted to strain ${\epsilon \;}^{j,k}\left(t\right)$ [%]. The initial resting length of all the sensors was set to 20 cm so that no sensor deflection occurred during all experiments. Then, second-order delay denoted by the differential Equation 5 was applied to ${\epsilon \;}^{j,k}\left(t\right)$ to obtain delayed strain ${\epsilon \;}_{\text{delay}}^{j,k}\left(t\right)$ . Due to the viscoelastic nature of soft material (Polygerinos et al., 2017), uniforming strain in a soft sensor requires time.

$$\frac{d^{2}\epsilon_{\text{delay}}\left(t\right)}{dt^{2}}+2\zeta \omega_{n}\frac{d\epsilon_{\text{delay}}\left(t\right)}{dt}+\omega_{n}^{2}\epsilon_{\text{delay}}\left(t\right)=K\omega_{n}^{2}\epsilon \left(t\right)$$ (5)

where $\omega_{n}$ is natural frequency, ζ is damping ratio, and K=1 is system gain. After that, a quadratic Equation 6 calculated change in resistance $\Delta R^{j,k}\left(t\right)$ from $\epsilon_{\text{delay}}^{j,k}\left(t\right)$ .

$$\Delta R\left(t\right)=b_{\text{quad}}\cdot \epsilon_{\text{delay}}^{2}\left(t\right)+c_{\text{quad}}\cdot \epsilon_{\text{delay}}\left(t\right)+d_{\text{quad}}$$ (6)

where $b_{\text{quad}},c_{\text{quad}},d_{\text{quad}}$ are constants. We set $b_{\text{quad}}=0$ and $c_{\text{quad}}=0$ to simplify parameter settings, as ΔR is always zero when ϵ=0 . This quadratic equation originated from Ohm’s law $R=\rho \frac{L}{S}$ where ρ,L,S are the resistivity, length, and the cross-section area of a soft sensor (Hegde et al., 2023). The literature (Shi et al., 2021) provides detailed derivation of Equation 6. Finally, sensor resistance R was obtained as follows (Equation 7):

$$R^{j,k}\left(t\right)=\Delta R^{j,k}\left(t\right)+R_{\text{baseline}}^{j,k}\left(t\right)$$ (7)

where $R_{\text{baseline}}^{j,k}\left(t\right)$ is the baseline sensor resistance. The obtained resistance values were processed with Gaussian noise with a standard deviation of 0.4% (Lin Y.-H. et al., 2023). Four parameters $b_{\text{quad}},\zeta ,\omega_{n},R_{\text{baseline}}$ were independently set for each sensor to represent their individual characteristic differences (Thuruthel et al., 2021; Sugiyama et al., 2021). We varied each parameter by ±20% from values that approximated experimental data found in the literature (Muth et al., 2014): $b_{\text{quad}}=1.189\times 10^{-2}$ , $\zeta =3.087\times 10^{-1}$ , $\omega_{n}=3.587$ rad/s, and $R_{\text{baseline}}=30.00$ k℧.

In addition to these nonlinearities, soft strain sensors often exhibit hysteresis and drift (Amjadi et al., 2016), which are challenging to model. In this paper, we ignored these complex temporal nonlinearities to simplify the modeling process. However, we already employed temporal nonlinearity as the second-order delay. In addition, the proposed framework consists of the LSTM and TDFNN, making it capable of modeling time-series data. Therefore, the framework is expected to address hysteresis and drift if it can handle the response delay. Moreover, researchers have succeeded in reducing sensor hysteresis and drift through sensor structure and material design (Park et al., 2019; Guan et al., 2023; Shen et al., 2022; Lu et al., 2020).

### 2.3 Training data collection

The musculoskeletal leg was actuated with motor babbling data. This process involves the iteration of ramping up/down and holding of input to allow the leg to explore its entire range of motion (Almanzor et al., 2024). The knee joint angle θ , muscle activation signals a , and muscle length l were recorded at a sampling frequency of 10 Hz. Then, each muscle length data was captured as sensor resistance values R . Consequently, the sensor sampling frequency was 10 Hz. During all the experiments, the resistance values obtained were processed using a low-pass filter with a cutoff frequency of 3 Hz. We generated 400 s of training data for the TDFNN and 1,600 s of training data for the LSTM. The training data were normalized and split into training and validation datasets in a ratio of 3:1.

The TDFNN dataset was augmented by zeroing all raw sensor resistance of randomly selected sensors. For each muscle, ten files containing the same 400 s of sensor resistance data were generated. Then, for each file, 0%, 10%, …, up to 90% of sensors were randomly selected and zeroed (i.e., masked). Since four muscles were involved, there are $2^{4}$ possible combinations whether each muscle contains masked sensors or not. Thus, the ten masked files of each muscle were combined. This process was followed by concatenating them with the corresponding masked or unprocessed sensor data from the other muscles. The concatenation was performed in $2^{4}-1$ combinations, excluding the case where no muscle contained masked data. As a result, the TDFNN dataset was composed of $10\times \left(2^{4}-1\right)=150$ files with different combinations of masked sensors.

### 2.4 Framework implementation

The stochastic LSTM had one LSTM layer with a size of 1,600 and a fully-connected layer for final output calculation. The mini-batch size was 8. ADAM optimizer with a learning rate of $5.0\times 10^{-5}$ was utilized. During the training process, the LSTM was trained using 150 steps of sequential data extracted from the dataset. The leg (muscle) and sensor responses included delay that can be modeled as first-order and second-order delay systems, respectively. The maximum time constant of muscle activation was 1.2 s, and the settling time of sensors was 2.0 s. Thus, the sequence length was set to 150, which is sufficiently higher than the total response delay (3.2 s). As a result, the LSTM effectively learned time-series characteristics between muscle activations and sensor responses. The loss function was calculated only with data at t≥50 . Typically, an initial sensor value is essential for the calculation of ${\hat{\mu }}_{t}$ and ${\hat{\sigma }}_{t}^{2}$ from the control input history. However, elasticity, common among soft actuators, gradually decreases the effect of their initial state. Therefore, by learning from data where the effect of the initial state was assumed to be sufficiently minimized, the LSTM was able to achieve the accurate estimation of ${\hat{\mu }}_{t}$ and ${\hat{\sigma }}_{t}^{2}$ without the initial state. The calculation threshold was set to 50, which was higher than the total response delay.

Regarding the TDFNN, the 1-D convolution and max pooling were carried out separately for each muscle and sample time steps. The kernel size was 3 with a stride of 1, and the output channels were 3. The pooling size and stride were 3. The max pooling results were concatenated into a vector and input to the fully-connected layer. The number of layers was 3, each with 500 hidden neurons. The input sequence length was set to 20 based on the sensor settling time of 2.0 s. The batch normalization layer was applied after the 1-D convolution layer to avoid overfitting. The mini-batch size was 512. ADAM optimizer with a learning rate of $1.0\times 10^{-4}$ was employed.

## 3 Results

### 3.1 Graceful degradation capability to different sensor degradation

First, we evaluated the fault detection and graceful degradation capabilities of the framework under diverse sensor degradation. We conducted five 100-s simulations using motor babbling inputs. For each trial, we generated five evaluation datasets: Normal (i.e., baseline), Lost, Stretch, Offset, and Deviation. These four degradation scenarios were designed to simulate diverse failure modes of soft sensors based on the literature. They effectively represented the fragility of soft sensors for framework evaluation. In each scenario, 30% of the sensors were randomly selected, and their readings were distorted as follows:

• *Lost*: Readings from subjected sensors were zeroed to simulate simple sensor loss (e.g., sensor rupture) (Lin Y.-H. et al., 2023; Thuruthel et al., 2019).
• *Stretch*: Sensor resistance values were recalculated with new sensor length $l_{\text{stretch}}$ randomly stretched up to 25%. This scenario comprehensively simulates changes in the geometric positioning of sensors, which can result from misalignment, environmental contact, or deformation of the proprioception target (Hegde et al., 2023; Lin Y.-H. et al., 2023).
• *Offset*: The persistent increase in baseline resistance, mainly caused by the self-healing process and plastic deformation of soft materials (Hardman et al., 2022; Terryn et al., 2021), was simulated. We increased the baseline resistance of subjected sensors by 50%.
• *Deviation*: The measured sensor resistance was randomly deviated by ±50% . Soft sensors experience diverse degradation and failures due to multiple factors (e.g., partial damage and healing, the effect of temperature, humidity, and sensor interference (Khatib et al., 2021; Porte et al., 2024)). This scenario provided a comprehensive simulation of these degradations, which are challenging to simulate uniformly with a single model.

In addition to this *Separate* dataset, we prepared a *Consecutive* dataset to evaluate the framework under actual deployment conditions, where degradation occurs consecutively during a single leg movement. To create the Consecutive dataset, we processed 100-s sensor readings, randomly selecting 30% of the sensors every 20 s to represent one of five states. The states followed the order: Normal, Lost, Stretch, Offset, and Deviation. We utilized the same five 100-s simulation data used for the Separate dataset. During the evaluation with the Consecutive dataset, the LSTM hidden states were not reset.

**FIGURE 5:**
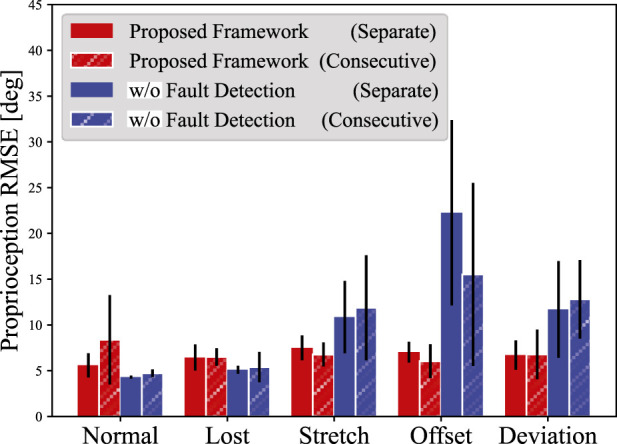
The average RMSEs of proprioception for each of the degradation scenarios. Five trials were conducted for both the Separate and Consecutive datasets. The error bar indicates the standard deviation. The blue bars show the proprioception results without the fault detection.

The stochastic LSTM and TDFNN were trained for 75 epochs and 30 epochs, respectively. Overfitting did not occur for both networks. Once trained, the networks did not experience any further retraining or intervention. Figure 5 shows the average root mean squared error (RMSE) of proprioception for five trials. The blue bar describes the result when all sensor data were directly input to the TDFNN without being zeroed. Table 2 lists the corresponding RMSEs. Note that for the calculation of the RMSE, the first 50 steps of the results were ignored as the LSTM training process did not use data at 0≤t≤50 . The results labeled as *w/o Fault Detection* represent the framework’s performance without fault detection. These results effectively simulate existing methods for soft sensor proprioception that do not include fault detection (see Section 1 for details). These results serve as a meaningful proxy for the evaluation with existing methods.

**TABLE 2:** Proprioception RMSE for different degradation scenarios.

		Proposed method [°]	Without fault detection [°]
Normal	Separate	5.59±1.32	4.30±0.17
	Consecutive	8.37±4.88	4.73±0.42
Lost	Separate	6.44±1.44	5.10±0.44
	Consecutive	6.50±0.95	5.40±1.65
Stretch	Separate	7.50±1.36	10.9±3.95
	Consecutive	6.77±1.31	11.9±5.73
Offset	Separate	7.03±1.14	22.3±10.1
	Consecutive	6.04±1.84	15.5±9.99
Deviation	Separate	6.71±1.61	11.7±5.28
	Consecutive	6.78±2.71	12.8±4.29

**FIGURE 6:**
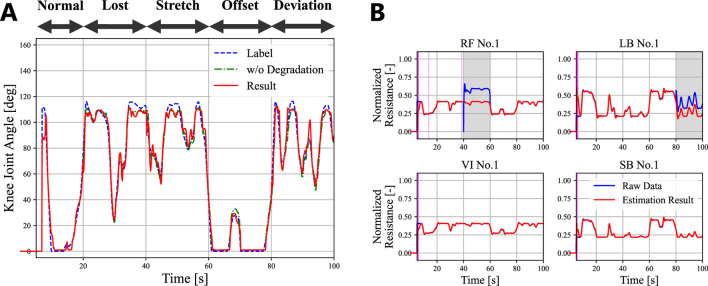
An example of proprioception with the Consecutive dataset. **(A)** The actual angle of the knee joint angle (blue dotted line), the result without degradation (green dash dot line), and the result with different degradation for every 20 s (red line). **(B)** The corresponding result of the fault detection for one of the sensors of each muscle. The blue line denotes the actual sensor readings. The red line and band denotes ${\hat{\mu }}_{t}$ and $3{\hat{\sigma }}_{t}$ , respectively. The grey vertical bands indicate successful fault detection, while the pink ones display false positives.

As shown in Figure 5, the proposed framework demonstrated graceful degradation and maintained accurate proprioception across all degradation scenarios and dataset types. For the Separate dataset, the average RMSEs remained nearly constant at 6.44° , 7.50° , 7.03° , and 6.71° for Lost, Stretch, Offset, and Deviation scenarios, respectively. Similarly, the Consecutive dataset resulted in a consistent trend, with RMSEs of 6.50° , 6.77° , 6.04° , and 6.78° for the corresponding scenarios. The differences in sensor degradation resulted in RMSE variations of only 1.06° and 0.74° , validating the effectiveness of the framework. In addition, Table 2 shows that the RMSE increase reached a maximum of 18.0° without fault detection, highlighting its importance. Figure 6 shows an example of proprioception conducted with the Consecutive dataset. Even if the degradation type was switched every 20 s, the proprioception RMSE was 5.92° , almost identical to the baseline RMSE of 5.24° . Figure 6B confirms that the LSTM performed precise fault detection with accurate sensor signal estimation. False positives at the beginning of the estimation followed by large ${\hat{\sigma }}_{t}^{2}$ are due to the LSTM training process. The LSTM could not perform estimation when the effect of the initial state did not decrease sufficiently. Subsequent small false positives of the RF sensor are due to noise.

### 3.2 Accuracy retention against increasing degraded sensors

Next, we evaluated the accuracy retention capability of the proposed framework, increasing the number of degraded sensors. Using the 100-s simulation data described in Section 3.1, we generated ten datasets for each trial by applying Offset degradation. In each of the ten datasets, 0%–90% of randomly selected sensors were degraded.

**FIGURE 7:**
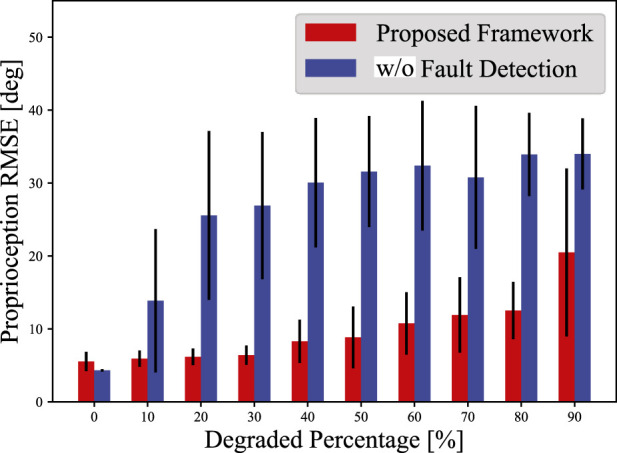
Proprioception RMSEs with different percentages of degraded sensors. The RMSE values are the average of five trials, and the error bars indicates the standard deviations. The blue bars show the proprioception results without fault detection.

Figure 7 and Table 3 presents the average proprioception RMSEs with different percentages of degraded sensors. The proposed framework tolerated the degradation in 50% of all sensors, with an average RMSE increase of only 3.30° . In contrast, without the fault detection component, even the 20% degradation led to the average RMSE increase of 21.3° , demonstrating the effectiveness of the proposed framework. When the percentage of degraded sensors exceeded 60%, the proprioception RMSE began to rise gradually. This increase is due to the lack of features for proprioception, as an input vector to the fully-connected layer contained more zeros. However, it is noteworthy that the RMSE increase was only 6.97° with the 80% loss of the sensors.

**TABLE 3:** Proprioception RMSE with increasing number of degraded sensors.

Degraded percentage [%]	0	10	20	30	40
Proposed Framework [%]	5.53±1.32	5.91±1.13	6.17±1.14	6.39±1.32	8.30±2.97
w/o Fault Detection [%]	4.31±0.18	13.9±9.83	25.6±11.6	26.9±10.1	30.1±8.87

Degraded percentage [%]	50	60	70	80	90
Proposed Framework [%]	8.83±4.23	10.8±4.29	11.9±5.17	12.5±3.93	20.5±11.5
w/o Fault Detection [%]	31.6±7.61	32.4±8.89	30.8±9.81	33.9±5.71	34.0±4.88

### 3.3 Scalability to different musculoskeletal configurations

Finally, the scalability of the proposed framework was evaluated. We prepared two additional models for the investigation, the RFLB and 6-muscles model (Figure 3). The RFLB model was developed as a proprioception target, incorporating both different actuation and sensor morphology. The 6-muscle model was designed as a proprioceptive target with significantly higher nonlinearity. As described in Figure 3, the RFLB model only included the RF and LB muscles to actuate the knee angle, while the number of sensors per muscle was doubled. On the other hand, the 6-muscles was a 2-DoF system with two states to perform proprioception (the hip and knee angle). Six muscles interacted with each other for the actuation. Moreover, the RF and LB muscles acted as bi-articular muscles. Consequently, the 6-muscles model had much higher system nonlinearity than the original model.

The same evaluations as in Section 3.1 were conducted using these two models. New networks were prepared and trained for each model. The stochastic LSTM/TDFNN were trained for 75/25 epochs for the RFLB model and 10/10 epochs for the 6-muscles model to avoid overfitting. The training data collection process, the other network’s parameter settings, and the evaluation procedure were the same as the original model, except for the TDFNN dataset for the RFLB model. We prepared three additional datasets to train the TDFNN effectively. Each dataset was created following the same procedure as the original model. Since there are $2^{2}$ combinations whether each muscle contains the degraded sensors or not, the TDFNN dataset consisted of $\left(1+3\right)\times \left(10\times \left(2^{2}-1\right)\right)=120$ files with different healthy/zeroed sensor combinations. Note that the TDFNN dataset for the 6-muscles model contained $10\times \left(2^{6}-1\right)=620$ files because the use of six muscles resulted in $2^{6}$ combinations of whether each muscle contains zeroed sensors or not.

**FIGURE 8:**
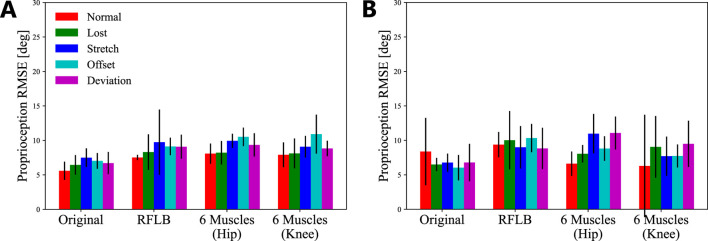
The average proprioception RMSEs with different musculoskeletal leg models. The error bars indicate the standard deviations of the five trials. **(A)** Separate dataset. **(B)** Consecutive dataset.

Figure 8 shows the evaluation results with the RFLB and 6-muscles model. Table 4 presents the corresponding proprioception RMSEs. For the RFLB model, while the baseline RMSE showed a slight increase, the proposed framework demonstrated graceful degradation comparable to that of the original model. In each scenario, the average RMSEs were 8.30° , 9.74° , 9.13° , and 9.08° for the Separate dataset and 10.0° , 9.00° , 10.3° , and 8.83° for the Consecutive dataset. RMSE variations remained within 1.44° or 1.47° . The maximum RMSE increase was only 2.22° in the Stretch scenario with the Separate dataset. With the 6-muscles model, reasonable proprioception RMSEs were maintained for all scenarios, with only a minimal increase in baseline RMSE. Across the four degradations, the average RMSE only changed by no more than 2.29° (hip joint) and 2.79° (knee joint) for the Separate dataset and 3.04° and 1.78° for the Consecutive dataset. Notably, the maximum RMSE increase was just 2.99° for the knee joint angle (Offset, the Separate dataset). While the Consecutive dataset resulted in prominent RMSE increases compared to the other models, the maximum increases were still limited to 4.49° and 3.20° (Deviation) despite the significant nonlinearity of the 6-muscles model.

**TABLE 4:** Proprioception RMSE with different leg models.

		RFLB [°]	6-muscles (Hip) [°]	6-muscles (Knee) [°]
Normal	Separate	7.52±0.39	8.08±1.46	7.91±1.80
	Consecutive	9.38±1.83	6.61±1.77	6.29±7.43
Lost	Separate	8.30±2.58	8.21±1.70	8.11±2.16
	Consecutive	10.0±4.23	8.06±1.27	9.05±4.47
Stretch	Separate	9.74±4.74	9.94±1.02	9.09±1.57
	Consecutive	9.00±3.08	11.0±2.86	7.71±2.84
Offset	Separate	9.13±1.26	10.5±1.34	10.9±2.83
	Consecutive	10.3±2.06	8.81±1.79	7.74±1.67
Deviation	Separate	9.08±1.75	9.35±1.69	8.83±1.12
	Consecutive	8.83±2.99	11.1±2.39	9.49±3.36

## 4 Discussions

### 4.1 Discussion

In this paper, we propose a novel learning-based graceful degradation framework for redundant soft sensor systems. For the first time, the proposed framework realizes graceful degradation for soft sensor proprioception against diverse sensor degradation scenarios.

We evaluated the proposed framework using a simulated musculoskeletal leg with soft sensors based on sufficiently nonlinear models. The soft sensor model was sufficiently reliable as it approximated the behavior of an experimentally verified soft sensor in the literature. This simulation allowed precise control over actual sensor degradation to evaluate the framework effectively. As a result, the experimental results demonstrated the framework’s excellent capability for graceful degradation, providing a solid understanding of its general behavior and performance. As shown in Figures 5, 6, 8, the framework adapted to four different sensor degradation scenarios and adequately retained the original proprioception accuracy. The fault detection with the stochastic LSTM was precise and essential for the framework’s graceful degradation. Under the Lost scenario, RMSEs were similar to those without fault detection as the scenario zeroed the values of affected sensors, resulting in identical original and processed sensor readings. In contrast, turning off the fault detection led to a significant RMSE increase in the Stretch, Offset, and Deviation scenarios. Particularly in the Offset scenario, sensor reading amplification due to degradation reduced proprioception accuracy. This is attributed to the max-pooling layer in the TDFNN, which caused amplified resistance values to affect the proprioception process directly. In the stretch scenario, the RMSE increase occurred for the same reason, while the minor amplification of sensor responses had less impact on the proprioception. In the Deviation scenario, RMSE increases were smaller than in Offset because the degradation both amplified and reduced sensor responses. When sensor responses were reduced, the max-pooling layer could exclude the affected readings from being input to the TDFNN, depending on the state of the proprioception target. Owing to the framework’s architecture, the proposed method successfully addressed both amplification and attenuation of sensor response. As a result, versatile graceful degradation was achieved across all the sensor failure modes. Additionally, Figure 7 shows noteworthy results that the framework could tolerate the deactivation of up to 80% of the constituent sensors. Furthermore, the experiments also revealed the scalability of the framework. Even when the proprioception target’s morphology and sensor configuration changed (RFLB model) or the system’s nonlinearity significantly increased (6-muscles model), the framework achieved graceful degradation across all degradation scenarios. Although small RMSE increases were observed for the evaluation with the 6-muscles model (the Consecutive dataset), this was due to the LSTM underfitting. The additional hip joint and bi-articular muscles of the 6-muscles model significantly complicated the forward healthy sensor model learned by the LSTM. Nevertheless, we did not change the hyperparameters and the amount of the LSTM dataset for comparison. As a result, the range of ±3σ (Equation 1) expanded, and false negatives impaired the accurate proprioception of the TDFNN (Supplementary Figure S2 in the supplemental material). Thus, fine-tuning the hyperparameters, such as Bayesian optimization (Akiba et al., 2019), will enhance the framework’s scalability.

Our work is distinguished from other existing research by its capability to tolerate diverse soft sensor degradation and availability for proprioception. Soft sensors have been widely modeled using learning-based approaches, and researchers have realized graceful degradation for soft sensor proprioception (Thuruthel et al., 2019; Wang et al., 2023). Researchers have also performed graceful degradation for soft sensor exteroception (Shih et al., 2020; Lo Preti et al., 2022; Dingley et al., 2023) and multimodal sensory systems (Zambelli et al., 2020; Chen et al., 2021; Lee et al., 2021; Liu et al., 2017; Zhi-Xuan et al., 2020; Wu and Goodman, 2018). However, these methods were not equipped with a fault detection component, and the evaluation scenarios were limited to complete sensor loss. On the other hand, soft sensors undergo various degradation due to their softness and nonlinearity (Terryn et al., 2021; Khatib et al., 2021; Porte et al., 2024; Terryn et al., 2022; Shen et al., 2016). Thus, direct input of distorted sensor readings will impair proprioception accuracy. Our framework employed fault detection based on the stochastic LSTM and tolerated diverse sensor degradation. The comparison with *w/o Fault Detection* results highlights significant improvements in our framework over existing approaches, particularly in Stretch, Offset, and Deviation scenarios. In addition, our complete framework also improved RMSE performance in the Lost scenario compared to existing methods, even though the evaluation setups were not identical. Specifically, our evaluation showed an RMSE increase of only 0.85° (0.61% of the leg joint’s range of motion), whereas existing literature (Thuruthel et al., 2019; Wang et al., 2023) reported RMSE increases exceeding 2.1% with 5% less sensor loss.

To our knowledge, no study has realized this capability for soft sensor proprioception. Fault detection has been applied to soft tactile sensing (Lo Preti et al., 2022) and multimodal sensing (Lee et al., 2021); however, signal-based or reconstruction-based fault detection utilized in these works is not suitable for proprioception. Due to the non-unique mapping illustrated in Figure 1, these approaches cannot determine whether the variation in sensor readings is caused by the deformation of the proprioception target or by sensor degradation, especially when the distortion in sensor readings is indistinct. In contrast, our LSTM-based fault detection avoided such a situation due to non-unique mapping by comparing sensor readings with the estimated responses of healthy sensors. Consequently, our framework achieved fault detection for proprioception against diverse sensor degradation. Furthermore, this fault detection and the subsequent zeroing process eliminated the need for seminal characterization and inclusion of all possible degradation to prepare the training dataset, typically required by the reconstruction approach (Lee et al., 2021; Zambelli et al., 2020). Despite the various types of degradation that soft sensors exhibit, our framework only requires its users to augment the obtained data by randomly zeroing sensor readings.

In summary, soft sensors are subjected to various types of degradation during operation. To address the degradation, the proposed graceful degradation framework for redundant soft sensors achieved consistently reliable proprioception. Unlike self-healing soft sensors, this framework realizes instantaneous adaptation to degradation without downtime or reduced sampling frequency. This capability allows soft robots to maintain proprioception under real-world deployments, where they experience repeated large deformations and various sources of damage. Due to their softness and nonlinearity, consistently reliable proprioception feedback is crucial for soft robots to retain performance. Therefore, the proposed framework will contribute significantly to enhancing the robustness of soft robots, maintaining their intelligent autonomy in real-world applications (e.g., soft grippers and rescue robots).

### 4.2 Limitations and future works

Our framework has three main directions for improvement: (1) adapting to damages and disturbances affecting the proprioception target itself, (2) evaluating the framework with an actual soft robot, and (3) further simplifying the training process.

**FIGURE 9:**
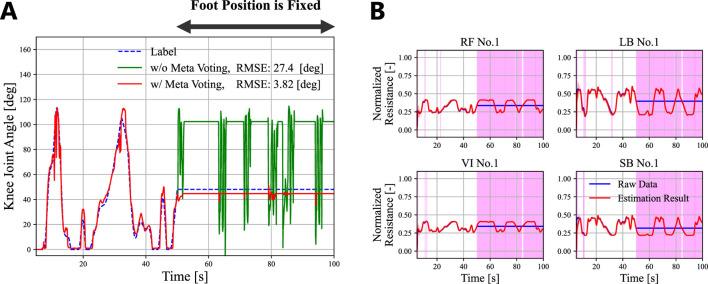
The contribution of the meta-voting algorithm to prevent external loads from affecting proprioception accuracy. **(A)** The actual angle of the knee joint angle (blue dotted line), the result without the meta-voting (green line), and the result with the meta-voting (red line). The foot position (i.e., the knee joint angle) was fixed at t=50 . **(B)** Examples of the corresponding LSTM outputs. The blue line denotes the actual sensor readings. The red line and band denotes ${\hat{\mu }}_{t}$ and $3{\hat{\sigma }}_{t}$ , respectively. The pink vertical bands display false positives.

With regard to the first point, the framework assumes that the proprioception target remains unaffected by damage to its body or external loads. The damages and disturbances can lead to sensor readings deviating from the estimated healthy values. As a result, all sensors are incorrectly identified as degraded, and proprioception accuracy will decrease. We will address this limitation by implementing a meta-voting algorithm that cancels sensor deactivation when the number of simultaneously degraded sensors exceeds a threshold. We conducted a preliminary experiment to investigate the effectiveness of this approach. While motor babbling input randomly actuated the Original model, we fixed the foot position (i.e., knee joint angle). We modified the proposed framework to cancel zeroing sensor readings if more than 50% of all sensors were simultaneously detected as degraded for five consecutive steps. As shown in Figure 9B, the LSTM output incorrect healthy sensor estimates after fixing the foot. Nevertheless, the FNN maintained accurate proprioception because the meta-voting canceled the incorrect sensor zeroing (Figure 9). In contrast, all sensors were zeroed without the meta-voting, and proprioception accuracy fell. We will further modify this algorithm so that the proposed framework can address the damages and external loads to a proprioception target. Furthermore, we will explore the use of tactile sensors in this modification (Lo Preti et al., 2022). By incorporating exteroceptive sensor data, the LSTM can account for the impact of damage and external loads on healthy sensor readings. As a result, the LSTM may directly compensate for these effects.

For future work (2), while we used a sufficiently nonlinear model in the simulation, we excluded hysteresis and drifts to simplify the modeling. Thus, evaluating the framework with an actual soft robot will highlight the framework’s practical effectiveness. Note that the simulation already incorporated temporal nonlinearity as a second-order delay. Additionally, the proposed framework consists of the LSTM and TDFNN. Hence, the framework can handle these nonlinearities through hyperparameter tuning.

Finally, regarding the third point, we augmented the training dataset by randomly zeroing sensor readings. However, this step can potentially be omitted by applying dropout before the 1-D convolution layer during training (see Figure 2). This minor adjustment will further simplify the training process.

## Data Availability

The raw data supporting the conclusions of this article will be made available by the authors, without undue reservation.

## References

[B1] Akiba T. Sano S. Yanase T. Ohta T. Koyama M. (2019). Optuna: a next-generation hyperparameter optimization framework, , 2623, 2631. 10.1145/3292500.3330701

[B2] Alatorre D. Axinte D. Rabani A. (2022). Continuum robot proprioception: the ionic liquid approach. IEEE Trans. Robotics 38, 526–535. 10.1109/TRO.2021.3082020

[B3] Almanzor E. Sugiyama T. Abdulali A. Hayashibe M. Iida F. (2024). Utilising redundancy in musculoskeletal systems for adaptive stiffness and muscle failure compensation: a model-free inverse statics approach. Bioinspir. Biomim. 19, 046015. 10.1088/1748-3190/ad5129 38806049

[B4] Almanzor E. Ye F. Shi J. Thuruthel T. G. Wurdemann H. A. Iida F. (2023). Static shape control of soft continuum robots using deep visual inverse kinematic models. IEEE Trans. Robotics 39, 2973–2988. 10.1109/TRO.2023.3275375

[B5] Amjadi M. Kyung K.-U. Park I. Sitti M. (2016). Stretchable, skin-mountable, and wearable strain sensors and their potential applications: a review. Adv. Funct. Mater. 26, 1678–1698. 10.1002/adfm.201504755

[B6] Cai G. Wang J. Qian K. Chen J. Li S. Lee P. S. (2017). Extremely stretchable strain sensors based on conductive self-healing dynamic cross-links hydrogels for human-motion detection. Adv. Sci. 4, 1600190. 10.1002/advs.201600190 PMC5323873 28251045

[B7] Carpenter M. B. (1968). The co-ordination and regulation of movements. J. Neuropathology Exp. Neurology 27, 348. 10.1097/00005072-196804000-00011

[B8] Chen K. Lee Y. Soh H. (2021). “Multi-modal mutual information (mummi) training for robust self-supervised deep reinforcement learning,” in 2021 IEEE international conference on robotics and automation (ICRA), 4274–4280. 10.1109/ICRA48506.2021.9561187

[B9] Chen Y. Guo S. Li C. Yang H. Hao L. (2018). Size recognition and adaptive grasping using an integration of actuating and sensing soft pneumatic gripper. Robotics Aut. Syst. 104, 14–24. 10.1016/j.robot.2018.02.020

[B10] Chou C.-P. Hannaford B. (1996). Measurement and modeling of mckibben pneumatic artificial muscles. IEEE Trans. Robotics Automation 12, 90–102. 10.1109/70.481753

[B11] Dingley G. Cox M. Soleimani M. (2023). Em-skin: an artificial robotic skin using magnetic inductance tomography. IEEE Trans. Instrum. Meas. 72, 1–9. 10.1109/TIM.2023.3268481 37323850

[B12] Driess D. Zimmermann H. Wolfen S. Suissa D. Haeufle D. Hennes D. (2018). “Learning to control redundant musculoskeletal systems with neural networks and sqp: exploiting muscle properties,” in 2018 IEEE international conference on robotics and automation (ICRA), 6461–6468. 10.1109/ICRA.2018.8463160

[B13] Erin O. Pol N. Valle L. Park Y.-L. (2016). “Design of a bio-inspired pneumatic artificial muscle with self-contained sensing,” in 2016 38th annual international conference of the IEEE engineering in medicine and biology society (EMBC), 2115–2119. 10.1109/EMBC.2016.7591146 28268749

[B14] Geyer H. Herr H. (2010). A muscle-reflex model that encodes principles of legged mechanics produces human walking dynamics and muscle activities. IEEE Trans. Neural. Syst. Rehabil. Eng. 18, 263–273. 10.1109/TNSRE.2010.2047592 20378480

[B15] Guan S. Xu C. Dong X. Qi M. (2023). A highly tough, fatigue-resistant, low hysteresis hybrid hydrogel with a hierarchical cross-linked structure for wearable strain sensors. J. Mater. Chem. A 11, 15404–15415. 10.1039/D3TA02584E

[B16] Haeufle D. Günther M. Bayer A. Schmitt S. (2014). Hill-type muscle model with serial damping and eccentric force–velocity relation. J. Biomechanics 47, 1531–1536. 10.1016/j.jbiomech.2014.02.009 24612719

[B17] Hardman D. Thuruthel T. G. Iida F. (2022). Self-healing ionic gelatin/glycerol hydrogels for strain sensing applications. NPG Asia Mater. 14, 11. 10.1038/s41427-022-00357-9

[B18] Hardman D. Thuruthel T. G. Iida F. (2023). Tactile perception in hydrogel-based robotic skins using data-driven electrical impedance tomography. Mater. Today Electron. 4, 100032. 10.1016/j.mtelec.2023.100032

[B19] Hegde C. Su J. Tan J. M. R. He K. Chen X. Magdassi S. (2023). Sensing in soft robotics. ACS Nano 17, 15277–15307. 10.1021/acsnano.3c04089 37530475 PMC10448757

[B20] Heinen F. Lund M. E. Rasmussen J. de Zee M. (2016). Muscle–tendon unit scaling methods of hill-type musculoskeletal models: an overview. Proc. Institution Mech. Eng. Part H J. Eng. Med. 230, 976–984. 10.1177/0954411916659894 27459500

[B21] Hill A. V. (1938). The heat of shortening and the dynamic constants of muscle. Proc. R. Soc. Lond. Ser. B, Biol. Sci. 126, 136–195. 10.1098/rspb.1938.0050 18152150

[B22] Hirashima M. Oya T. (2016). How does the brain solve muscle redundancy? filling the gap between optimization and muscle synergy hypotheses. Neurosci. Res. 104, 80–87. 10.1016/j.neures.2015.12.008 26724372

[B23] Kawaharazuka K. Nishiura M. Toshimitsu Y. Omura Y. Koga Y. Asano Y. (2022). Robust continuous motion strategy against muscle rupture using online learning of redundant intersensory networks for musculoskeletal humanoids. Robotics Aut. Syst. 152, 104067. 10.1016/j.robot.2022.104067

[B24] Khatib M. Zohar O. Haick H. (2021). Self-healing soft sensors: from material design to implementation. Adv. Mater. 33, 2004190. 10.1002/adma.202004190 33533124

[B25] Kim D. Kim S.-H. Kim T. Kang B. B. Lee M. Park W. (2021). Review of machine learning methods in soft robotics. PLOS ONE 16, e0246102. 10.1371/journal.pone.0246102 33600496 PMC7891779

[B26] Lee M. A. Tan M. Zhu Y. Bohg J. (2021). Detect, reject, correct: crossmodal compensation of corrupted sensors. IEEE International Conference on Robotics and Automation ICRA, 909–916. 10.1109/ICRA48506.2021.9561847

[B27] Li C. Cui Y.-L. Tian G.-L. Shu Y. Wang X.-F. Tian H. (2015). Flexible cnt-array double helices strain sensor with high stretchability for motion capture. Sci. Rep. 5, 15554. 10.1038/srep15554 26530904 PMC4632108

[B28] Lin Y.-H. Siddall R. Schwab F. Fukushima T. Banerjee H. Baek Y. (2023a). Modeling and control of a soft robotic fish with integrated soft sensing. Adv. Intell. Syst. 5, 2000244. 10.1002/aisy.202000244

[B29] Lin Z. Wang Z. Zhao W. Xu Y. Wang X. Zhang T. (2023b). Recent advances in perceptive intelligence for soft robotics. Adv. Intell. Syst. 5, 2200329. 10.1002/aisy.202200329

[B30] Liu G.-H. Siravuru A. Prabhakar S. Veloso M. Kantor G. (2017). “Learning end-to-end multimodal sensor policies for autonomous navigation,” in Proceedings of the 1st annual conference on robot learning. PMLR), vol. 78 of *Proceedings of machine learning research* . Editors Levine S. Vanhoucke V. Goldberg K., 249–261.

[B31] Lo Preti M. Totaro M. Falotico E. Crepaldi M. Beccai L. (2022). Online pressure map reconstruction in a multitouch soft optical waveguide skin. IEEE/ASME Trans. Mechatronics 27, 4530–4540. 10.1109/TMECH.2022.3158979

[B32] Lu S. Chen D. Hao R. Luo S. Wang M. (2020). Design, fabrication and characterization of soft sensors through egain for soft pneumatic actuators. Measurement 164, 107996. 10.1016/j.measurement.2020.107996

[B33] Marques H. G. Bharadwaj A. Iida F. (2014). From spontaneous motor activity to coordinated behaviour: a developmental model. PLoS Comput. Biol. 10, e1003653. 10.1371/journal.pcbi.1003653 25057775 PMC4109855

[B34] Masuda H. Hitzmann A. Hosoda K. Ikemoto S. (2019). Common dimensional autoencoder for learning redundant muscle-posture mappings of complex musculoskeletal robots. IEEE/RSJ International Conference on Intelligent Robots and Systems IROS, 2545–2550. 10.1109/IROS40897.2019.8968605

[B35] Mattmann C. Clemens F. Tröster G. (2008). Sensor for measuring strain in textile. Sensors 8, 3719–3732. 10.3390/s8063719 27879904 PMC3714661

[B36] Mazzolai B. Mondini A. Dottore E. D. Margheri L. Carpi F. Suzumori K. (2022). Roadmap on soft robotics: multifunctionality, adaptability and growth without borders. Multifunct. Mater. 5, 032001. 10.1088/2399-7532/ac4c95

[B37] Meyer A. J. Patten C. Fregly B. J. (2017). Lower extremity emg-driven modeling of walking with automated adjustment of musculoskeletal geometry. PLOS ONE 12, 0179698–e179724. 10.1371/journal.pone.0179698 PMC5507406 28700708

[B38] Murata S. Namikawa J. Arie H. Sugano S. Tani J. (2013). Learning to reproduce fluctuating time series by inferring their time-dependent stochastic properties: application in robot learning via tutoring. IEEE Trans. Aut. Ment. Dev. 5, 298–310. 10.1109/TAMD.2013.2258019

[B39] Muth J. T. Vogt D. M. Truby R. L. Mengüç Y. Kolesky D. B. Wood R. J. (2014). Embedded 3d printing of strain sensors within highly stretchable elastomers. Adv. Mater. 26, 6307–6312. 10.1002/adma.201400334 24934143

[B40] Nguyen N. H. Ho V. A. (2022). Mechanics and morphological compensation strategy for trimmed soft whisker sensor. Soft Robot. 9, 135–153. 10.1089/soro.2020.0056 33464996 PMC8885438

[B41] Park K. Kim S. Lee H. Park I. Kim J. (2019). Low-hysteresis and low-interference soft tactile sensor using a conductive coated porous elastomer and a structure for interference reduction. Sensors Actuators A Phys. 295, 541–550. 10.1016/j.sna.2019.06.026

[B42] Park Y.-L. Chen B.-R. Wood R. J. (2012). Design and fabrication of soft artificial skin using embedded microchannels and liquid conductors. IEEE Sensors J. 12, 2711–2718. 10.1109/JSEN.2012.2200790

[B43] Philipp R. Ohta N. Takayama Y. Hara Y. Funato T. Seki K. (2023). “Neural adaptation in response to a tendon cross-union of an antagonistic muscle pair in the forearm of the macaque: an emg and egog study,” in *The 46th annual Meeting of the Japan neuroscience society* (the Japan neuroscience society), 3Pa–042.

[B44] Polygerinos P. Correll N. Morin S. A. Mosadegh B. Onal C. D. Petersen K. (2017). Soft robotics: review of fluid-driven intrinsically soft devices; manufacturing, sensing, control, and applications in human-robot interaction. Adv. Eng. Mater. 19, 1700016. 10.1002/adem.201700016

[B45] Porte E. Eristoff S. Agrawala A. Kramer-Bottiglio R. (2024). Characterization of temperature and humidity dependence in soft elastomer behavior. Soft Robot. 11, 118–130. 10.1089/soro.2023.0004 37669451 PMC10880277

[B46] Proske U. Gandevia S. C. (2012). The proprioceptive senses: their roles in signaling body shape, body position and movement, and muscle force. Physiol. Rev. 92, 1651–1697. 10.1152/physrev.00048.2011 23073629

[B47] Roels E. Terryn S. Brancart J. Sahraeeazartamar F. Clemens F. Van Assche G. (2022). Self-healing sensorized soft robots. Mater. Today Electron. 1, 100003. 10.1016/j.mtelec.2022.100003

[B48] Shen Z. Yi J. Li X. Lo M. H. P. Chen M. Z. Q. Hu Y. (2016). A soft stretchable bending sensor and data glove applications. Robotics Biomimetics 3, 22. 10.1186/s40638-016-0051-1 28003951 PMC5133288

[B49] Shen Z. Zhang Z. Zhang N. Li J. Zhou P. Hu F. (2022). High-stretchability, ultralow-hysteresis conductingpolymer hydrogel strain sensors for soft machines. Adv. Mater. 34, 2203650. 10.1002/adma.202203650 35726439

[B50] Shi C. Zhao Y. Zhu P. Xiao J. Nie G. (2021). Highly stretchable and rehealable wearable strain sensor based on dynamic covalent thermoset and liquid metal. Smart Mater. Struct. 30, 105001. 10.1088/1361-665X/ac1b3a

[B51] Shih B. Lathrop E. Adibnazari I. Martin R. Park Y.-L. Tolley M. T. (2020). “Classification of components of affective touch using rapidly-manufacturable soft sensor skins,” in 2020 3rd IEEE international conference on soft robotics (RoboSoft), 182–187. 10.1109/RoboSoft48309.2020.9116023

[B52] Shintake J. Piskarev Y. Jeong S. H. Floreano D. (2018). Ultrastretchable strain sensors using carbon black-filled elastomer composites and comparison of capacitive versus resistive sensors. Adv. Mater. Technol. 3, 1700284. 10.1002/admt.201700284

[B53] Souri H. Banerjee H. Jusufi A. Radacsi N. Stokes A. A. Park I. (2020). Wearable and stretchable strain sensors: materials, sensing mechanisms, and applications. Adv. Intell. Syst. 2, 2000039. 10.1002/aisy.202000039

[B54] Sugiyama T. Kutsuzawa K. Owaki D. Hayashibe M. (2021). Individual deformability compensation of soft hydraulic actuators through iterative learning-based neural network. Bioinpir. Biomim. 16, 056016. 10.1088/1748-3190/ac1b6f 34359064

[B55] Sugiyama T. Kutsuzawa K. Owaki D. Hayashibe M. (2024). Latent representation-based learning controller for pneumatic and hydraulic dual actuation of pressure-driven soft actuators. Soft Robot. 11, 105–117. 10.1089/soro.2022.0224 37590488 PMC10880272

[B56] Terryn S. Hardman D. Thuruthel T. G. Roels E. Sahraeeazartamar F. Iida F. (2022). Learning-based damage recovery for healable soft electronic skins. Adv. Intell. Syst. 4, 2200115. 10.1002/aisy.202200115

[B57] Terryn S. Langenbach J. Roels E. Brancart J. Bakkali-Hassani C. Poutrel Q.-A. (2021). A review on self-healing polymers for soft robotics. Mater. Today 47, 187–205. 10.1016/j.mattod.2021.01.009

[B58] Thuruthel T. G. Gilday K. Iida F. (2020). “Drift-free latent space representation for soft strain sensors,” in 2020 3rd IEEE international conference on soft robotics (RoboSoft), 138–143. 10.1109/RoboSoft48309.2020.9116021

[B59] Thuruthel T. G. Hughes J. Georgopoulou A. Clemens F. Iida F. (2021). Using redundant and disjoint time-variant soft robotic sensors for accurate static state estimation. IEEE Robotics Automation Lett. 6, 2099–2105. 10.1109/LRA.2021.3061399

[B60] Thuruthel T. G. Shih B. Laschi C. Tolley M. T. (2019). Soft robot perception using embedded soft sensors and recurrent neural networks. Sci. Robotics 4, eaav1488. 10.1126/scirobotics.aav1488 33137762

[B61] Wang H. Totaro M. Beccai L. (2018). Toward perceptive soft robots: progress and challenges. Adv. Sci. 5, 1800541. 10.1002/advs.201800541 PMC6145216 30250796

[B62] Wang L. Lam J. Chen X. Li J. Zhang R. Su Y. (2023). Soft robot proprioception using unified soft body encoding and recurrent neural network. Soft Robot. 10, 825–837. 10.1089/soro.2021.0056 37001175

[B63] Wang Y. Gong S. Wang S. J. Simon G. P. Cheng W. (2016). Volume-invariant ionic liquid microbands as highly durable wearable biomedical sensors. Mater. Horiz. 3, 208–213. 10.1039/C5MH00284B

[B64] Wu M. Goodman N. (2018). Multimodal generative models for scalable weakly-supervised learning. Proc. 32nd Int. Conf. Neural Inf. Process. Syst. 18, 5580–5590. 10.48550/arXiv.1802.05335

[B65] Xu S. Vogt D. M. Hsu W.-H. Osborne J. Walsh T. Foster J. R. (2019). Biocompatible soft fluidic strain and force sensors for wearable devices. Adv. Funct. Mater. 29, 1807058. 10.1002/adfm.201807058 31372108 PMC6675035

[B66] Yamada T. Yamamoto Y. H. Y. Yomogida Y. Izadi-Najafabadi A. Futaba D. N. Hata K. (2011). A stretchable carbon nanotube strain sensor for human-motion detection. Nat. Nanotechnol. 6, 296–301. 10.1038/nnano.2011.36 21441912

[B67] Yang H. Ding S. Wang J. Sun S. Swaminathan R. Ng S. W. L. (2024). Computational design of ultra-robust strain sensors for soft robot perception and autonomy. Nat. Commun. 15, 1636. 10.1038/s41467-024-45786-y 38388467 PMC10883982

[B68] Yasa O. Toshimitsu Y. Michelis M. Y. Jones L. S. Filippi M. Buchner T. (2023). An overview of soft robotics. Annu. Rev. Control, Robotics, Aut. Syst. 6, 1–29. 10.1146/annurev-control-062322-100607

[B69] Zambelli M. Cully A. Demiris Y. (2020). Multimodal representation models for prediction and control from partial information. Robotics Aut. Syst. 123, 103312. 10.1016/j.robot.2019.103312

[B70] Zhi-Xuan T. Soh H. Ong D. (2020). Factorized inference in deep markov models for incomplete multimodal time series. Proc. AAAI Conf. Artif. Intell. 34, 10334–10341. 10.1609/aaai.v34i06.6597

