# Supplementary material for: Versatile graceful degradation framework for bio-inspired proprioception with redundant soft sensors
Source: Front Robot AI. 2025 Jan 6;11:1504651. doi: 10.3389/frobt.2024.1504651 (PMC11743178; doi:10.3389/frobt.2024.1504651)
Supplement: Supplementary file 1 [file Supplementaryfile1.pdf]

Supplementary Material

1 RELATED WORKS

1.1 The Use of Self-healing Soft Materials to Retain Reliable Sensing

As review papers describe (Khatib et al., 2021; Terryn et al., 2021), self-healing materials have been employed to address the fragility issue of soft sensors. Importantly, researchers have retained sensing accuracy throughout the damage-healing cycles of these sensors. Terryn et al. (2022) proposed a deep learning-based approach for modeling self-healable soft electronic skins. The researchers utilized a deep feedforward neural network to compensate for the temporal nonlinearities of the sensors, resulting in accurate tactile sensing. Following the damage and healing processes, offline transfer learning techniques were applied to recover sensing accuracy. Consequently, the original sensing accuracy was recovered with fewer than 200 recalibration data points. Notably, a similar recalibration strategy has also been used to manage sensor degradations in redundant sensor configurations (Kawaharazuka et al., 2022).

However, most self-healing materials require time, ranging from minutes to hours, to complete the healing process. This feature leads to downtime and low sensing frequency (Yang et al., 2024). Moreover, the healing process will change sensor properties, necessitating recalibration to recover proprioception accuracy after degradation. In contrast, redundant soft sensor configuration enables to achieve instant adaptation to sensor failures without downtime or intervention (e.g., recalibration (Roels et al., 2022), reconfiguration of sensor positions (Nguyen and Ho, 2022)). Such systems achieve graceful degradation through redundancy by compensating for the failed sensors using the remaining healthy ones.

1.2 Examples on Learning-based Graceful Degradation

This section introduces some examples of graceful degradation leveraging learning-based approaches, particularly neural networks. First, as presented in the introduction, Thuruthel et al. (2019) realized accurate multimodal sensing of a soft continuum actuator by combining a redundant sensor configuration (three embedded soft sensors and one pressure sensor) with a Long Short-Term Memory (LSTM) network. In addition to their main contribution, the researchers demonstrated that the LSTM network adapted to the virtual loss of one or two embedded soft sensors and retained proprioception accuracy in the simulation experiment. As another example, Wang et al. (2023) parallelly arranged redundant soft pneumatic chambers as pressure receptors to measure angles of a soft continuum joint. Then, the LSTM was utilized to model joint kinematics with unified pressure information of the actuators and receptors. Due to the redundant configuration, graceful degradation was achieved, and proprioception error (root mean squared error, RMSE) barely increased when one or two receptors were lost.

Some researchers also have realized graceful degradation for soft tactile sensing with distributed sensor arrays (Shih et al., 2020; Lo Preti et al., 2022; Dingley et al., 2023) and even for a redundant sensing system consisting of multiple sensory modalities (i.e., tolerating missing sensory modalities) (Zambelli et al., 2020; Chen et al., 2021; Lee et al., 2021; Liu et al., 2017; Zhi-Xuan et al., 2020; Wu and Goodman, 2018). As an example of soft tactile sensing, Shih et al. (2020) developed a soft sensor skin array from liquid metal-embedded silicone elastomer. The LSTM was trained to distinguish various tactile cues from sensor readings. After LSTM training, a 2x2 sensor array could classify ten types of tactile sensation with 97% accuracy. Moreover, the researchers analyzed the graceful degradation capability of the array, and the classification accuracy was maintained with only a 25 to 36% reduction when one of the arrays was

removed. Regarding a multimodal sensor system, Zambelli et al. (2020) proposed a multimodal variational autoencoder for the sensorimotor capability of a humanoid robot. Proprioceptive (i.e., joint angles), visual, tactile, sound, and motor command data were utilized for sensory modalities. The proposed autoencoder was able to reconstruct missing sensory modalities, predict the robot's sensorimotor states and visual trajectories of another agent, and imitate the observed trajectory of another agent. The training dataset for the autoencoder included multiple combinations of missing modalities. Notably, even when the input was limited to visual data alone, the increase in joint angle reconstruction RMSE was less than 1% compared to that with the complete set of input modalities.

In these studies, graceful degradation was achieved. The developed methods tolerated the loss of constituent sensors, achieving consistently reliable proprioception or tactile/multimodal sensing with retained accuracy. However, the evaluations were limited to the complete loss of sensors. Soft sensors will undergo diverse degradation scenarios due to their softness and nonlinear behavior (Terryn et al., 2021; Khatib et al., 2021; Porte et al., 2024; Terryn et al., 2022; Shen et al., 2016). On the other hand, these methods did not include sensor fault detection components and directly received failed (i.e., zeroed) sensor readings. Thus, non-zero but distorted sensor input, which is significantly different from those in training data, will significantly affect the network performance and decline the proprioception accuracy. Furthermore, in case a graceful degradation framework requires pre-training of degradation patterns or combinations as in the literature (Zambelli et al., 2020), the diverse and unpredictable soft sensor degradation makes the training data acquisition process infeasible.

In contrast, our proposed framework incorporates fault detection based on healthy sensor responses estimated from control input. This fault detection approach detects and zeros degraded soft sensor readings to prevent a proprioception network from receiving distorted signals. Due to this architecture, our proposed method achieves graceful degradation of soft sensor proprioception, tolerating various sensor degradation patterns.

1.3 Graceful Degradation with Sensor Fault Detection

Some researchers have developed a graceful degradation framework with fault detection components for soft tactile sensing and multimodal sensor data fusion. For example, Lo Preti et al. (2022) proposed an online reconstruction method to map pressures on soft optical waveguide skin. Twenty-four photoemitters and 24 photoreceivers surrounded the skin, and a time-delay feedforward neural network (TDFNN) was employed for the mapping. Signals from five adjacent healthy photoreceivers were equally weighted so that the five contributions sum to one before being sent to the TDFNN. The researchers implemented a built-in fault-tolerant mechanism that monitors the response histories of the photoreceivers. When the response varies higher than 20% of the standard deviation of the history, the weight for corresponding photoreceivers is reduced to 0.05. Although the evaluation was limited to the complete loss of photoreceivers, the increase in pressure mapping error was less than 10%, even when half of them were lost. However, such signal monitoring-based fault detection is unsuitable for soft sensor proprioception. As explained in Section 1, the non-unique mapping issue of the soft sensors makes it infeasible to distinguish whether the signal variation is due to a deformation of a proprioception target or the degradation of sensors. Thus, the direct application of signal-based approaches to soft sensor proprioception will cause numerous false positives and negatives.

Lee et al. (2021) proposed a data fusion framework for a multimodal sensing system with built-in sensor error detection and graceful degradation capability. The researchers developed a Crossmodal Compensation Model (CCM), which encodes multimodal sensor data into a latent representation z_{mult}, to generate stable state feedback for learned policy rollout, compensating for distorted sensor inputs. The CCM monitors

reconstruction error from z_{mult} for each sensor modality. If the error exceeds a threshold, the corresponding sensor modality is rejected to encode a new latent representation z'_{mult} only using the other modalities. Only the modality with the largest error deviation was excluded to inhibit degradation in one sensor modality from affecting the reconstruction of the others. The CCM was trained to minimize the L2 distance between z_{mult} and z'_{mult}. As a result, the CCM compensated for various types of degradation (e.g., tilting and random occlusion of visual data, random Gaussian noise addition to force/torque signals), and a target policy which received z'_{mult} achieved graceful degradation with an average success rate of 79 to 82$\%$ in executing peg-in-hole tasks. Nevertheless, those reconstruction approaches require extensive pre-training of degradation patterns for accurate fault detection in soft sensor proprioception. Hence, the diversity and unpredictable nature of soft sensor degradation will make the training data acquisition infeasible. Moreover, the non-unique mapping would affect the fault detection as sensor degradation might be overlooked if the distorted sensor readings are similar to healthy sensor readings in different states of a target. As a result, proprioception accuracy can decrease.

Apart from these examples, a comprehensive review on sensor fault detection (Gao et al., 2015a,b) introduces four general approaches and their combinations (i.e., hybrids approaches): (1) model-based, (2) signal-based, (3) history-based (data-driven), and (4) active diagnosis. As explained, signal-based approaches are unsuitable for soft sensor proprioception due to non-unique mapping problems. History-based approaches are based on monitoring the consistency between observed sensor behavior and underlying knowledge extracted from a large volume of historical data. It is also challenging to apply this approach for proprioception because it is required to characterize and collect all possible sensor degradation in advance. Regarding active diagnosis, which injects test signals at preset intervals, this approach makes instant adaptation to sensor degradations (i.e., graceful degradation) impossible. Other than these approaches, fault detection through comparing each sensor (e.g., majority voting) is also unavailable due to the unfixed geometry and individual characteristic differences of soft materials (Gao et al., 2015a; Sugiyama et al., 2021).

From these backgrounds, we employed a model-based approach for fault detection. As obtaining a sensor model that includes all possible degradation patterns is challenging, we utilized a healthy forward model (i.e., a model that outputs responses of healthy sensors from control input to a proprioception target). The proposed framework performs fault detection by comparing sensor readings with the healthy estimates obtained from control inputs. Owing to this architecture, we realized the detection of diverse soft sensor degradation while avoiding the problems related to non-unique mapping and the acquisition of training datasets.

2 SUPPLEMENTARY TABLES AND FIGURES

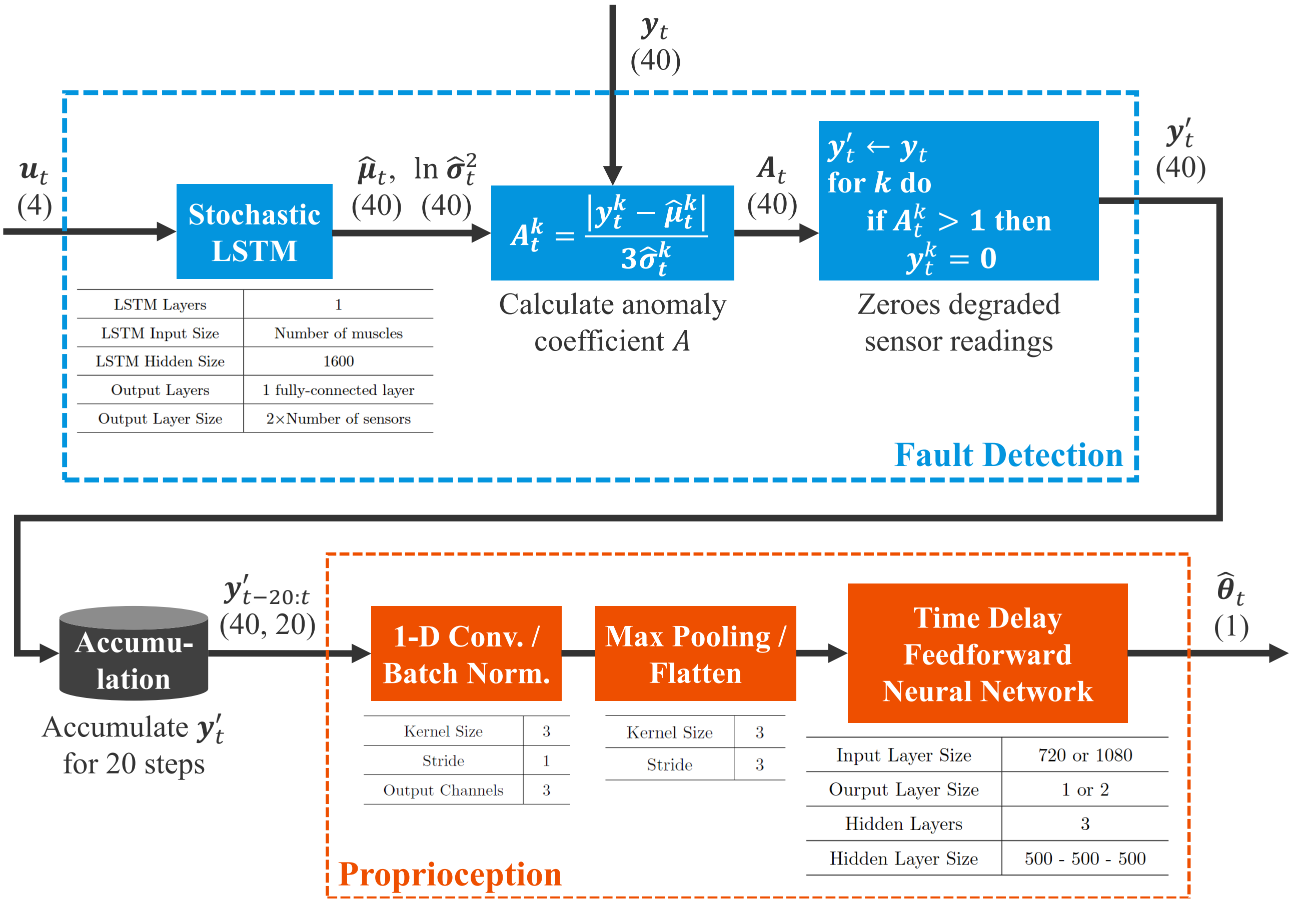

Figure S1. The detailed flow of the proposed framework at time step t. The stochastic LSTM is responsible for fault detection, while the TDFNN performs proprioception. The numbers below each variable represent their respective dimensions when the original model is used. The variables in the figure are defined as follows: k represents the sensor number, $\boldsymbol{u}$ denotes control inputs, $\boldsymbol{y}$ corresponds to sensor readings, $\hat{\boldsymbol{\mu}}$ is the estimated mean, $\hat{\boldsymbol{\sigma}}^2$ is the estimated variance, A represents the anomaly coefficient, $\boldsymbol{y}'$ refers to processed sensor readings, and $\hat{\boldsymbol{\theta}}$ indicates the estimated states. The input/output layer sizes of the TDFNN are 720/1 for the original and RFLB models, and 1080/2 for the 6-muscle model.

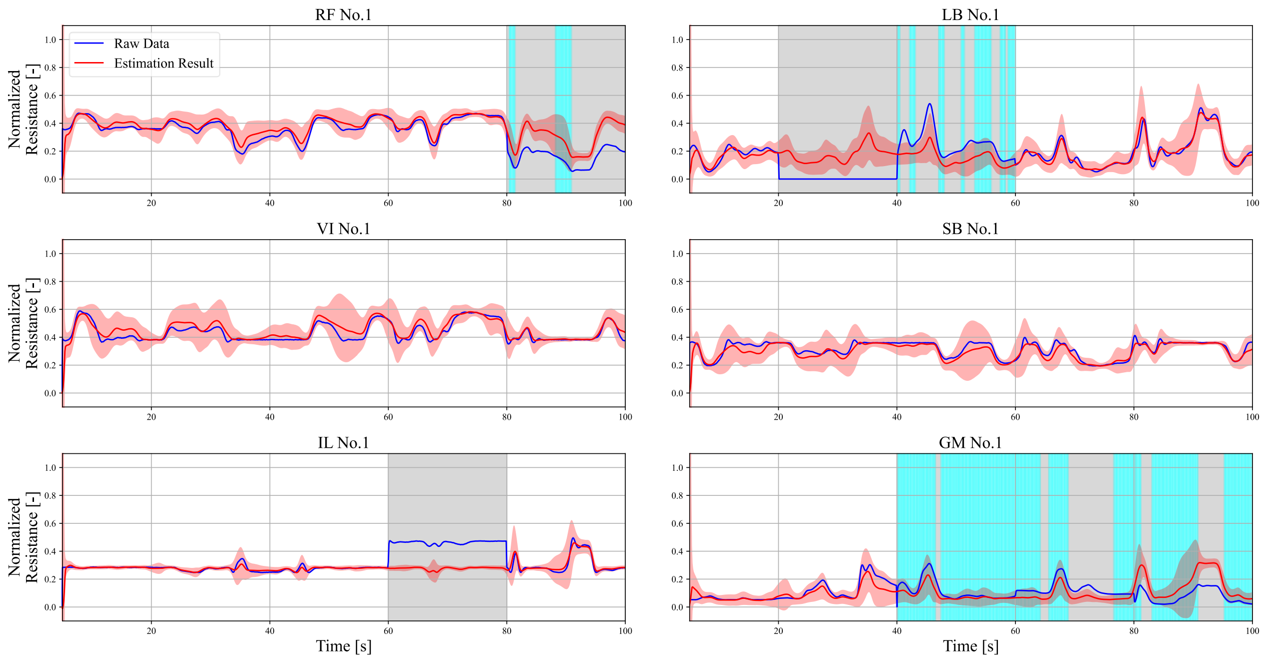

Figure S2. An example of fault detection for the 6-muscles model. The first sensor of each muscle is shown. One trial of the Consecutive dataset was utilized to obtain this result. The blue line denotes the actual sensor readings. The red line and band denotes $\hat{\mu}_t$ and $3\hat{\sigma}_t$, respectively. The grey vertical bands indicate successful fault detection, while the cyan ones display false negatives. Compared to Fig. 6B in the manuscript, the range of $\pm 3\hat{\sigma}_t$ is wider, and false negatives occurred due to the underfitting of the LSTM.

REFERENCES

Chen, K., Lee, Y., and Soh, H. (2021). Multi-modal mutual information (mummi) training for robust self-supervised deep reinforcement learning. In *2021 IEEE International Conference on Robotics and Automation (ICRA)*. 4274–4280. doi:10.1109/ICRA48506.2021.9561187

Dingley, G., Cox, M., and Soleimani, M. (2023). Em-skin: An artificial robotic skin using magnetic inductance tomography. *IEEE Transactions on Instrumentation and Measurement* 72, 1–9. doi:10.1109/TIM.2023.3268481

Gao, Z., Cecati, C., and Ding, S. X. (2015a). A survey of fault diagnosis and fault-tolerant techniques—part i: Fault diagnosis with model-based and signal-based approaches. *IEEE Transactions on Industrial Electronics* 62, 3757–3767. doi:10.1109/TIE.2015.2417501

Gao, Z., Cecati, C., and Ding, S. X. (2015b). A survey of fault diagnosis and fault-tolerant techniques—part ii: Fault diagnosis with knowledge-based and hybrid/active approaches. *IEEE Transactions on Industrial Electronics* 62, 3768–3774. doi:10.1109/TIE.2015.2419013

Kawaharazuka, K., Nishiura, M., Toshimitsu, Y., Omura, Y., Koga, Y., Asano, Y., et al. (2022). Robust continuous motion strategy against muscle rupture using online learning of redundant intersensory networks for musculoskeletal humanoids. *Robotics and Autonomous Systems* 152, 104067. doi:https://doi.org/10.1016/j.robot.2022.104067

Khatib, M., Zohar, O., and Haick, H. (2021). Self-healing soft sensors: From material design to implementation. *Advanced Materials* 33, 2004190. doi:https://doi.org/10.1002/adma.202004190

Lee, M. A., Tan, M., Zhu, Y., and Bohg, J. (2021). Detect, reject, correct: Crossmodal compensation of corrupted sensors. In *2021 IEEE International Conference on Robotics and Automation (ICRA)*. 909–916. doi:10.1109/ICRA48506.2021.9561847

Liu, G.-H., Siravuru, A., Prabhakar, S., Veloso, M., and Kantor, G. (2017). Learning end-to-end multimodal sensor policies for autonomous navigation. In *Proceedings of the 1st Annual Conference on Robot Learning*, eds. S. Levine, V. Vanhoucke, and K. Goldberg (PMLR), vol. 78 of *Proceedings of Machine Learning Research*, 249–261

Lo Preti, M., Totaro, M., Falotico, E., Crepaldi, M., and Beccai, L. (2022). Online pressure map reconstruction in a multitouch soft optical waveguide skin. *IEEE/ASME Transactions on Mechatronics* 27, 4530–4540. doi:10.1109/TMECH.2022.3158979

Nguyen, N. H. and Ho, V. A. (2022). Mechanics and morphological compensation strategy for trimmed soft whisker sensor. *Soft Robotics* 9, 135–153. doi:10.1089/soro.2020.0056

Porte, E., Eristoff, S., Agrawala, A., and Kramer-Bottiglio, R. (2024). Characterization of temperature and humidity dependence in soft elastomer behavior. *Soft Robotics* 11, 118–130. doi:10.1089/soro.2023.0004

Roels, E., Terryn, S., Brancart, J., Sahraeeazartamar, F., Clemens, F., Van Assche, G., et al. (2022). Self-healing sensorized soft robots. *Materials Today Electronics* 1, 100003. doi:https://doi.org/10.1016/j.mtelec.2022.100003

Shen, Z., Yi, J., Li, X., Lo, M. H. P., Chen, M. Z. Q., Hu, Y., et al. (2016). A soft stretchable bending sensor and data glove applications. *Robotics and Biomimetics* 3. doi:10.1186/s40638-016-0051-1

Shih, B., Lathrop, E., Adibnazari, I., Martin, R., Park, Y.-L., and Tolley, M. T. (2020). Classification of components of affective touch using rapidly-manufacturable soft sensor skins. In *2020 3rd IEEE International Conference on Soft Robotics (RoboSoft)*. 182–187. doi:10.1109/RoboSoft48309.2020.9116023

Sugiyama, T., Kutsuzawa, K., Owaki, D., and Hayashibe, M. (2021). Individual deformability compensation of soft hydraulic actuators through iterative learning-based neural network. *Bioinspir. Biomim.* 16, 056016. doi:10.1088/1748-3190/ac1b6f

Terryn, S., Hardman, D., Thuruthel, T. G., Roels, E., Sahraeeazartamar, F., and Iida, F. (2022). Learning-based damage recovery for healable soft electronic skins. *Adv. Intell. Syst.* 4, 2200115. doi:https://doi.org/10.1002/aisy.202200115

Terryn, S., Langenbach, J., Roels, E., Brancart, J., Bakkali-Hassani, C., Poutrel, Q.-A., et al. (2021). A review on self-healing polymers for soft robotics. *Materials Today* 47, 187–205. doi:https://doi.org/10.1016/j.mattod.2021.01.009

Thuruthel, T. G., Shih, B., Laschi, C., and Tolley, M. T. (2019). Soft robot perception using embedded soft sensors and recurrent neural networks. *Science Robotics* 4, eaav1488. doi:10.1126/scirobotics.aav1488

Wang, L., Lam, J., Chen, X., Li, J., Zhang, R., Su, Y., et al. (2023). Soft robot proprioception using unified soft body encoding and recurrent neural network. *Soft Robotics* 10, 825–837. doi:10.1089/soro.2021.0056

Wu, M. and Goodman, N. (2018). Multimodal generative models for scalable weakly-supervised learning. In *Proceedings of the 32nd International Conference on Neural Information Processing Systems* (Red Hook, NY, USA: Curran Associates Inc.), NIPS'18, 5580–5590

Yang, H., Ding, S., Wang, J., Sun, S., Swaminathan, R., Ng, S. W. L., et al. (2024). Computational design of ultra-robust strain sensors for soft robot perception and autonomy. *Nat. Commun.* 15. doi:https://doi.org/10.1038/s41467-024-45786-y

Zambelli, M., Cully, A., and Demiris, Y. (2020). Multimodal representation models for prediction and control from partial information. *Robotics and Autonomous Systems* 123, 103312. doi:https://doi.org/10.1016/j.robot.2019.103312

Zhi-Xuan, T., Soh, H., and Ong, D. (2020). Factorized inference in deep markov models for incomplete multimodal time series. *Proceedings of the AAAI Conference on Artificial Intelligence* 34, 10334–10341. doi:10.1609/aaai.v34i06.6597
